# A content validated questionnaire for assessment of self reported venous blood sampling practices

**DOI:** 10.1186/1756-0500-5-39

**Published:** 2012-01-19

**Authors:** Karin Bölenius, Christine Brulin, Kjell Grankvist, Marie Lindkvist, Johan Söderberg

**Affiliations:** 1 Department of Nursing, Umeå University, Umeå, Sweden; 2 Department of Medical Biosciences, Clinical Chemistry, Umeå University, Umeå, Sweden; 3 Department of Statistics, Umeå University, Umeå, Sweden; 4 Department of Nursing, Umeå University, 901 87 Umeå, Sweden

**Keywords:** Error risk assessment, Patient safety, Preanalytical errors, Questionnaires, Reliability and validity, Risk, Venous blood sampling

## Abstract

**Background:**

Venous blood sampling is a common procedure in health care. It is strictly regulated by national and international guidelines. Deviations from guidelines due to human mistakes can cause patient harm. Validated questionnaires for health care personnel can be used to assess preventable "near misses"--i.e. potential errors and nonconformities during venous blood sampling practices that could transform into adverse events. However, no validated questionnaire that assesses nonconformities in venous blood sampling has previously been presented. The aim was to test a recently developed questionnaire in self reported venous blood sampling practices for validity and reliability.

**Findings:**

We developed a questionnaire to assess deviations from best practices during venous blood sampling. The questionnaire contained questions about patient identification, test request management, test tube labeling, test tube handling, information search procedures and frequencies of error reporting. For content validity, the questionnaire was confirmed by experts on questionnaires and venous blood sampling. For reliability, test-retest statistics were used on the questionnaire answered twice. The final venous blood sampling questionnaire included 19 questions out of which 9 had in total 34 underlying items. It was found to have content validity. The test-retest analysis demonstrated that the items were generally stable. In total, 82% of the items fulfilled the reliability acceptance criteria.

**Conclusions:**

The questionnaire could be used for assessment of "near miss" practices that could jeopardize patient safety and gives several benefits instead of assessing rare adverse events only. The higher frequencies of "near miss" practices allows for quantitative analysis of the effect of corrective interventions and to benchmark preanalytical quality not only at the laboratory/hospital level but also at the health care unit/hospital ward.

## Background

During recent years there has been an intense world-wide debate on the inadequate patient safety in health care [1]. In Sweden alone, it has been estimated that annually approx. 100 000 patients suffer from injuries, nearly 10 000 receives lasting injuries and 3000 die due to mistakes in health care [2]. Studies in other countries show similar figures [1,3-5]. To note is that most injuries are caused by human errors [1-3]. It is therefore important to develop routines and systems to increase patient safety and promote cost effectiveness [2,3,6].

A number of studies suggest that most of the errors in laboratory medicine are linked to the preanalytical phase i.e. before the sample is analyzed in a laboratory. Analytical errors (within the laboratory) and post-analytical errors (reporting and interpretation of results) are less frequent [4,7-10].

Venous blood sampling (VBS) is complex and demands both knowledge and skill [11]. VBS is a frequent procedure in health care [8] and strictly regulated by similar national and international standards [11-14]. The majority of errors in VBS are due to human mistakes which in several cases cause patient harm [4,7]. Errors that can occur during VBS and handling are numerous, and include inaccurate patient and sample identification, inadequately filled tubes [7,9,11], specimen haemolysis [8,9,11,15], and improper handling of anticoagulants [9,11,12]. Standards that refer to best preanalytical practice [11-14] aim to reduce or alleviate many of these errors. In Sweden VBS is usually decentralized and performed by enrolled nurses, registered nurses and biomedical technicians [14]. The VBS standards are not always followed and interventions are needed to ensure patient safety and reduce risk due to VBS errors [15-21]. Our previous studies have investigated practices of VBS staff in hospital wards and primary health care centers (PHCs) by means of a self reported venous blood sampling questionnaire (VBSQ) [16-21]. The initial VBSQ was developed, further modified and used in several studies [16-21].

Questionnaire surveys have several benefits as they are practical to handle, self-administered, economical and give the respondents anonymity. It is also easy to reach a large study group in a large geographic area [22]. When using questionnaires it is important to confirm validity (i.e. how well an instrument measures what it is supposed to measure) [22-24] and reliability (i.e. stability) of the included items [22,23]. However, no validated VBSQ has previously been published. The aim was to test a recently developed questionnaire on self reported venous blood sampling practices for validity and reliability.

## Materials and methods

### Study design

The VBSQ (Additional file 1) was designed in cooperation with a clinical chemist and instructors at the Clinical Chemistry Laboratory, Umea University Hospital and researchers at the Dept of Nursing and Dept of Medical Biosciences, Umea University. The VBSQ was based on standard instructions for VBS [13,14] in line with international recommendations [11,12]. The questionnaire was in a Swedish version and tested for content, face validity and reliability in a Swedish health care context. The layout of the questionnaire was designed to be easy to read and number of pages was limited, to ensure that the questionnaire could be completed within a reasonable period of time.

### Setting

This follow-up study was performed in the PHC and hospital settings in northern Sweden.

### Population

#### Validity

To achieve content validity an extensive dialogue with professionals on different aspects on VBS and questionnaire design was performed. This included senior researchers, registered nurses, enrolled nurses, biomedical technicians, heads of wards, physicians at University Departments and hospital wards as well as researchers with considerable experience in questionnaire design. Instructors from the local laboratory also double-checked that questions were in accordance with guidelines [13,14]. Criterion validity is applied as we followed guidelines. This is verified as laboratory staff perform more correct VBS according to guidelines [25-27], and therefore can be used to benchmark VBS performance, which is also confirmed in our studies [16-18,20,21]. As there are no other questionnaires that can be used as golden standard, concurrent and predictive criterion validity is not applicable [24]. To establish face validity, a focus group with seven enrolled nurses discussed the VBSQ at two occasions. They had considerable experience of VBS in different clinical settings, both in hospitals and in PHCs. In Sweden VBS are most often performed by enrolled nurses, therefore enrolled nurses match as a focus group. Their role was to identify any item that could possibly be misinterpreted. All gave consent to participate in the study.

#### Reliability

The test and establishment of VBSQ (Additional file 1) stability was conducted in the PHCs setting after the validity process. The head of the PHCs assisted in the distribution and gave permission to perform the survey. Thirty one enrolled nurses, registered nurses, and biomedical technicians of PHCs in two County Councils in northern Sweden were engaged in the test-retest study. Three participants did not answer the VBSQ in the follow up, thus 28 participants were included in the analyses and gave written informed consent to participate in the study. Of these, 64% performed VBS every day, 25% every week and 11% every month or more seldom. The median age was 52 years (Q1 = 44; Q3 = 58), and the median employment time was 7 years, (Q1 = 2; Q3 = 19).

### Instrument

Overall the questionnaire was developed to assess practical tasks in accordance with national guidelines [14]. The individual questions were not related to each other i.e., exploratory factor analysis is not applicable. The VBSQ included background characteristics (6 questions), patient identification and collection of specimen (4 questions; 10 items), sample storage and seeking information (2 questions; 7 items) test request management and test-tube labeling (4 questions; 12 items) frequency of error reporting and suggestions (3 questions; 8 items), (Additional file 1). One question was answered yes/no, while the majority were answered by a four-point scale; Never, Seldom, Often, and Always. All "by other mean" items (n = 4) were excluded from the test-retest due to high internal missing rate but were retained in the questionnaire. A careful instruction containing an illustration on how to complete the questionnaire was located on the front page. It is to note that it was clearly pointed out that the respondents were to state how they usually performed VBS practices and not how they knew it was to be performed.

### Procedures and data analysis

#### Validity

Content validation differs from other forms of validity testing. It is not based on the scores from the questionnaire but from discussions between experts whether the questionnaire instrument appears logical to this group of experts [24]. The development and analysis of questions and items of the VBSQ was followed and transcribed by members in the research group. During the questionnaire development, in focus group and experts discussions, extensive efforts were made to ensure that each item was clearly formulated, easy to understand, and could not be misinterpreted. The focus group of enrolled nurses read, answered the questions, and discussed the questions and the items on the VBSQ. The project member wrote down their opinions and amended the questionnaire with help of other project members including instructors from the laboratory. It was also ensured that the questions and items still were according to the guidelines. Thereafter the focus group members reconsidered the questionnaire and had a renewed reflected discussion with project members.

#### Reliability

The VBSQ was distributed at two different occasions with 3-4 weeks between the test and retest. Data was analyzed by using three measures, Spearman's rank correlation (rs), Kappa coefficient (K) and percentage agreement (%A) in the persons answer. Non-symmetrical K values were manually counted by the author [28]. The items were accepted if they passed at least one of two set criteria [22,29];

• Criterion one: K **≥ **0.61 = good **or **rs **≥ **0.7 **or **%A **≥ **90%

• Criterion two: K ≥ 0.51 = moderate **and **rs ≥ 0.6 **or **K ≥ 0.51 = moderate **and **%A ≥ 80%

Criterion two complemented criterion one in those cases where a combination of the used measures were judged to be acceptable for acceptance of the items. SPSS 18.0 for Windows (SPSS Inc., Chicago, IL) was used for analysis.

### Ethics

This study was approved by the Regional Ethical Review Board in Umeå (Dnr 06-104 M). The VBSQ were kept in a locked space and all questionnaires were decoded. Only the researchers had access to the codes and the corresponding names. The participants were assured confidentiality by the information letter and also that they could withdraw from the survey at any time without declaring any reason. They were also informed that data would be presented at group level only.

## Results

The process of establishing validity resulted in several modifications. The answer alternatives were reduced from five to four (the alternative sometimes was removed) and several questions was also specifically modified to suit the PHCs setting. Focus group meetings resulted in removal of a question about order of test tube collection. A few questions were rephrased and further modified. Each question and items was controlled to be easy to understand and clearly outlined. The number of included questions was judged to be possible to complete in a reasonable amount of time. The final VBSQ included 19 questions out of which 9 had in total 34 underlying items. Thereafter, content validity of the VBSQ was judged as acceptable by experts in VBS and questionnaire design and face validity was established by the focus group with vast experience. Criterion validity was applied as we followed guidelines. This is verified as laboratory staff performs more correct VBS according to guidelines.

When analyzing the VBSQ for reliability, the K and rs of the test-retest varied between high and low which indicated that some of the items needed to be modified or excluded. In total, 82% of the questions with underlying items fulfilled the reliability acceptance criteria (Table 1). The questions or items of criterion one was fulfilled by 71% and criterion two by 68%. Items that did not fulfill the acceptance criteria were 7c, 8b, 8c, 10a, 16a, 18e and 18f. VBSQ items 8b, 8c, and 16a were not stable but important to retain because of the items being interrelated with other underlying items.

**Table 1:** Stability and agreement for the test-retest (n = 28). Removed items are marked into bold font and cursive, retained items marked only cursive.

Question	n-cat^a^	n (%)^b^	r_S_^c^	*κ^d^*	%A^e^	1^f^	2^g^
**7 How and how often do you check the identity of a patient when collecting venous blood sampling?**							
a) I ask the patient to state his/her name...	2/4	28 (100)	0.63	0.62	86%	Ok	Ok
b) I already know the patient...	3/4	27 (96)	0.89	0.71	85%	Ok	Ok
**c) I ask the patients relatives**	**4/4**	**24 (86)**	**0.54**	**0.38**	**67%**		
d) I check the patient's ID-card	4/4	28 (100)	0.81	0.69	82%	Ok	Ok
**8 If you use stasis when performing venous blood sampling, when do you remove it?**							
a) Before the first...	4/4	27 (96)	0.72	0.31	48%	Ok	
*b) During sampling*	*4/4*	*26 (93)*	*0.58*	*0.46*	*61%*		
*c) After the sampling is finished*	*4/4*	*26 (93)*	*0.53*	*0.45*	*69%*		
d) If there is difficulty collecting...	4/4	26 (93)	0.72	0.71	81%	Ok	Ok
**9 How long do you usually allow your patient to rest**	6/6	28 (100)	0.87	0.78	82%	Ok	Ok
**10 How often do you carry out the following tasks?**							
*a) If the test tube has an additive*...	*3/4*	*28 (100)*	*0.45*	*0.47*	*82%*		
b) Use an automatic test tube inverter	4/4	26 (93)	0.89	0.89	92%	Ok	Ok
**11 What do you do when you are not sure how a sample should be collected?**							
a) I check printed instructions...	4/4	25 (89)	0.76	0.69	84%	Ok	Ok
b) I check the instructions (internal network)...	4/4	25 (89)	0.96	0.87	92%	Ok	Ok
c) Ask a colleague	4/4	26 (93)	0.83	0.61	73%	Ok	Ok
d) I call the lab	4/4	27 (96)	0.82	0.67	85%	Ok	Ok
**12 How do you store the test tubes immediately after sampling?**							
a) Lying on a workbench or...	4/4	24 (86)	0.78	0.53	83%	Ok	Ok
b) In the pocket...	2/4	24 (86)	1.00	1.00	100%	Ok	Ok
c) In a test-tube stand	4/4	26 (93)	0.99	0.84	92%	Ok	Ok
**13 How often does someone else mark the sampling time on the test request?**	3/5	27 (96)	0.65	0.53	81%		Ok
**14 When do you mark the sampling time on the test request, if you do it yourself?**	2/5	26 (93)	0.92	0.91	96%	Ok	Ok
**15 How often do you perform the following tasks?**							
a) Compare the patient's name and...	2/4	28 (100)	0.56	0.47	93%	Ok	
b) Use test request that somebody...	4/4	28 (100)	0.69	0.57	75%		Ok
c) Sign the test request	2/4	27 (96)	0.69	0.65	96%	Ok	Ok
d) Check the information...	4/4	27 (96)	0.77	0.57	93%	Ok	Ok
e) Adjust sampling time...	4/4	25 (89)	0.69	0.68	80%	Ok	Ok
f) Check that the test request...	4/4	27 (96)	0.70	0.52	78%	Ok	Ok
**16 When do you label the test tube?**							
*a) Before I approach the patient*	*4/4*	*28 (100)*	*0.37*	*0.33*	*68%*		
b) Alongside the patient before...	4/4	27 (96)	0.77	0.33	52%	Ok	
c) Alongside the patient after...	4/4	28 (100)	0.77	0.52	64%	Ok	Ok
d) At a later occasion	2/4	27 (96)	0.69	0.65	96%	Ok	Ok
e) Somebody else has labelled the test tube in advance	3/4	27 (96)	0.49	0.52	88%	Ok	Ok
f) Somebody else labels the test tube after sampling	2/4	28 (100)	0.56	0.47	93%	Ok	
**17 Approximately, how many error reports have you written after observing or making an error in venous blood sampling?**	2/2	26 (93)	1.00	1.00	100%	Ok	Ok
**18 If you have refrained from writing an error report: What was/were the reason/reasons?**							
a) I don't have enough time	4/4	25 (89)	0.75	0.49	64%	Ok	
b) It wouldn't make any difference	4/4	24 (86)	0.77	0.65	79%	Ok	Ok
c) Nobody else does	3/4	24 (86)	0.77	0.65	83%	Ok	Ok
d) It is too complicated	3/4	23 (82)	0.65	0.56	74%		Ok
**e) The head of the PHC writes the error reports**	**3/4**	**24 (86)**	**0.42**	**0.28**	**71%**		
**f) I am concerned about possible consequences**	**2/4**	**21 (75)**	**0.09**	**-0.07**	**81%**		
**19 To what extent do you agree in the following statements?**							
a) I have enough knowledge...	2/4	28 (100)	0.59	0.58	86%		Ok
b) Proper collection and handling...	3/4	26 (93)	0.53	0.63	81%	Ok	Ok

^a^n-cat, number of answered categories^, b^n(%), numbers of respondents ^c^r_s_, Spearmans rangcorrelation ^d^*κ*, Kappacoefficient, ^e^%A, Numbers of agreements in%, ^f ^Criteria1, *κ ≥ *0.61 or r_s _≥ 0.7 or%A ≥ 90%, ^g ^Criteria 2, *κ ≥ 0.51 and *r_s _≥ 0.6 or *κ ≥ 0.51 and *%A ≥ 80%.

## Discussion

The low preanalytical error rates noted by the individual laboratory calls for large databases and appropriate techniques for the detection of errors and their consequent reduction [7]. Comparisons of error rates and the effect of interventions have hitherto been possible (although rarely performed) only between laboratories and not between individual hospital wards or PHCs. Most preanalytical procedures that increase the risks for or lead to adverse effects that jeopardize patient safety are performed locally at PHCs and hospital wards [30]. Detailed analyses of occurring errors and risks, present an opportunity to work preventively [31]. To assess risk of near-misses would allow for general and directed corrective educational interventions and also permit comparison and benchmarking of preanalytical practices between wards and PHCs. Thus, focusing on improving the frequency of near misses would thus lead to better opportunities for quality improvement than mere focus on assessment of underreported incidents or registered rare adverse errors [32]. Methods to assess frequently occurring, error-prone everyday tasks at the hospital ward/PHC level are therefore urgently needed. Questionnaire surveys have as far as we know not previously been used to assess how VBS staff follows recommended preanalytical guideline practices in order to improve patient safety. However, this study will give researchers and practitioners a trustworthy VBSQ to survey and/or evaluate VBS practices.

We developed and used the questionnaire to assess practical performance of VBS of health care personnel [16-21]. Generally, questionnaire surveys are cost effective and easy to coordinate [22] and in our studies we were able to survey almost all VBS personnel distributed throughout the County Council.

In this study, we found that the VBSQ had content validity (determined what it was intended to assess) as both content validity including face validity and criterion validity were judged to be reasonable.

VBSQ were based on standards for VBS and our studies demonstrate that laboratory staff can be used as benchmark in comparison of preanalytical practices [16-18,20,21]. VBS by laboratory staff results in fewer haemolyzed samples than VBS by non laboratory staff [25]. Results from previous surveys showed better performance by the laboratory staff indicate some degree of Criterion validity. Construct validity was not tested since this kind of validity was judged not to be applicable to the investigated questionnaire.

The VBSQ stability determined by the reliability coefficient of the test-retest varied and indicated that some items had to be removed or reformulated. Overall, the stability in the questionnaire was acceptable as 71% of the items fulfilled the acceptance criterion one and 68% criterion two, and 82% fulfilled either of the criteria. However, answers in questionnaires can be affected by memory interference and by changes in knowledge and behavior over time, which influence the stability (test-retest) of the measure. The recommended interval between test and retest is 2 days to 2 weeks [24]. We used an interval of 3-4 weeks so higher stability could have been reached with shorter interval. It is to note that the stability of the items were high considering that we surveyed how VBS was actually performed and not knowledge of how it should be performed according to guidelines. A weakness of using the K for stability assessment is that it takes no account to the degree of disagreement [29]. Thus, complementing with percentage agreement in the persons answer when K is low and rs high is an aid in the stability acceptance decision. For example, in item 12a (low K, high rs and high percentage) and for 15a with (low K, low rs and high percentage) actually only two respondents changed their answers. Items 7c, 18e, 18f will be removed from future VBSQ surveys because of lack of stability. Item 10a will be reformulated due to unexpected dual interpretations by surveyed VBS staff.

The most stable questions were those where VBS were not situation-dependent and the answer therefore expected to reflect the actual practices (example question 12). In contrast patient-related questions such as question 8 when to remove stasis are dependent of the VBS situation and less stable. Items 18e and 18f covering error reporting practices had surprising low rs,%A and K. The loss of respondents was 25%. A few responders wrote that error reports were not an ordinary work tasks and were unable to respond. The VBS personnel are obliged to follow the Swedish National Board of Health and Welfare constitutions [33] which are the basis for the Handbook for health care [14] and for local directives [13]. Also, every staff member has a duty to write error reports [33], therefore question 18 is important to retain but to reformulate.

This VBSQ (Additional file 1) was developed, managed and validated in the Swedish health care context. In other health care settings, with differences such as language, culture and working conditions, necessitates renewal of validity and stability testing of the VBSQ. In particular, performing stability testing with test-retest has an important bearing on questionnaire development prior to surveys and follow-up of interventions.

According to the WHO, a patient safety incident is an event or circumstance that could have resulted, or did result, in unnecessary harm to a patient [34]. Also, ISO technical specification stresses the need of a recurrent review of laboratory non-conformities, errors and incidents, as well as improving the quality of laboratory services and patient safety. The use of reliable quality indicators that effectively evaluate the quality of the steps of the preanalytical phase can thus drive improvement programs for better laboratory services and patient safety [10].

## Conclusions

To conclude, the questionnaire has acceptable validity and reliability and could be used for assessment of "near miss" practices that could jeopardize patient safety. After removal of non-stable questions the questionnaire finally contained 19 questions out of which 9 had in total 34 underlying items. The retained non-stable items 8b, 8c, 10a, 16a should be reformulated and thereafter re-validated. A reliable questionnaire to assess "near miss" practices offers several benefits over assessing rare adverse events only. Iterated questionnaire surveys of hospital wards and PHCs VBS staff practices would high-light specific problems and make it possible to follow the effect of corrective actions. Such information is of outmost importance in order to eliminate "near miss" events by error prevention.

## Abbreviations

VBS: Venous blood sampling; VBSQ: Venous blood sampling questionnaire; PHC: Primary health care centre.

## Competing interests

The authors declare that they have no competing interests.

## Authors' contributions

KB, JS, CB and KG proposed the original idea for the study and developed and modified the questionnaire, initially designed also by Olof Wallin. KB, CB and ML executed the statistical analyses. KB drafted the manuscript and all the authors read the manuscript critically for important intellectual content and approved the final manuscript.

## Supplementary Material

Additional file 1 **Questionnaire regarding blood-sampling practices at primary health care centres (PHCs)**. Click here for file
