# Supplementary material for: A content validated questionnaire for assessment of self reported venous blood sampling practices
Source: BMC Res Notes. 2012 Jan 19;5:39. doi: 10.1186/1756-0500-5-39 (PMC3342148; doi:10.1186/1756-0500-5-39)
Supplement: Additional file 1 — Questionnaire regarding blood-sampling practices at primary health care centres (PHCs). [file 1756-0500-5-39-S1.DOC]

**Questionnaire regarding blood-sampling practices at primary health care centres (PHCs)**

(Please read the attached information letter before proceeding below)

Instructions for completing the questionnaire

This questionnaire contains questions concerning venous blood sampling for clinical chemistry analysis. You will be asked to complete a few yes/no questions. You will also be asked if you *Never, Seldom, Often* or *Always* carry out a task in a specific manner. It is important that you select the most suitable alternative from the choices given. This means that you should not mark an answer between two alternatives. In the final question we will ask for your opinion and suggestions.

*Mark your answers by placing a cross in the box beside the most suitable alternative* ☒

*If you wish to change an answer, fill in the incorrectly marked box completely* █

We are interested in how you carry out your daily work. If you are unsure of how to answer a question – answer how you usually do.

**In questions like in the example below, it is IMPORTANT that you mark one alternative for EACH row.**

Example of a correct answer:

**Ⓐ** Which utensil do you use for eating soup?

|  | Never | Seldom | Often | Always |
| --- | --- | --- | --- | --- |
| a) Fork | ☒ | □ | □ | □ |
| b) Spoon | □ | □ | □ | ☒ |
| c) Knife | ☒ | □ | □ | □ |

Place the completed questionnaire in the enclosed envelope, seal it and give the envelope to the head of your PHC. We will collect the envelopes at the end of the survey period.

*It is crucial that you answer the questions as honestly and carefully as possible. This means that you should answer how you* ***usually*** *perform a given task, not how you know that it is supposed to be done.*

The following section contain questions regarding year of birth, education and routines

1 In which year were you born?

19 ­_ _

2 For how long have you been

employed at this PHC?

_ _ year _ _ month

3 How often do you carry out venous

blood sampling?

| Every workday | □ |
| --- | --- |
| Every week | □ |
| Every month | □ |
| More seldom | □ |
| Never | □ |

4 Which year did you receive your most recent education in venous blood sampling?

| _ _ _ _ |  |
| --- | --- |
|  |  |

5 Which year did you before that, receive education for venous blood sampling?

_ _ _ _

6 Do you want education in venous

blood sampling?

| No | □ |
| --- | --- |
| Yes | □ |

The following questions concern patient identification and collection of specimen

*It is important for you to mark ONE alternative for EACH row in the following section.*

7 How and how often do you check the identity of a patient when collecting venous blood samples?

|  | Never | Seldom | Often | Always |
| --- | --- | --- | --- | --- |
| **a)** I ask the patient to state his/her name and social security  number | □ | □ | □ | □ |
| **b)** I already know the patient and don’t have to check this | □ | □ | □ | □ |
| **~~c)~~** ~~I~~ ~~ask the patients relatives~~ | ~~□~~ | ~~□~~ | ~~□~~ | ~~□~~ |
| **d)** I check the patient’s photo-ID | □ | □ | □ | □ |
| **e)** By other means:................................................................ | □ | □ | □ | □ |

8 If you use stasis when performing venous blood sampling, when do you remove it?

|  | Never | Seldom | Often | Always |
| --- | --- | --- | --- | --- |
| **a)** Before the first sample is drawn | □ | □ | □ | □ |
| **b)** During sampling | □ | □ | □ | □ |
| **c)** When the sampling is finished | □ | □ | □ | □ |
| **d)** If there is difficulty collecting the sample, I keep the stasis  as long as necessary | □ | □ | □ | □ |

9 How long do you usually allow your patient to rest (supine or sitting) prior to venous blood sampling?

| Not at all | 0-5 min | 6-10 min | 11-15 min | More then 15 min | I don’t check this |
| --- | --- | --- | --- | --- | --- |
| □ | □ | □ | □ | □ | □ |

10 How often do you carry out the following tasks?

|  | Never | Seldom | Often | Always |
| --- | --- | --- | --- | --- |
| **a)** If the test tube has an additive, invert the test tube several  times immediately before the next one is filled? | □ | □ | □ | □ |
| **b)** Use an automatic test tube inverter | □ | □ | □ | □ |

The following questions concern sample storage and seeking information

*It is important for you to mark ONE alternative for EACH row in the following section.*

11 What do you do when you are not sure of how a sample should be collected?

|  | Never | Seldom | Often | Always |
| --- | --- | --- | --- | --- |
| **a)** I check printed sampling instructions (available at the PHC)  issued by the lab | □ | □ | □ | □ |
| **b)** I check sampling instructions on the lab homepage/on the  internal network | □ | □ | □ | □ |
| **c)** I ask a college | □ | □ | □ | □ |
| **d)** I call the lab | □ | □ | □ | □ |
| **e**) By other way:..................................................................... | □ | □ | □ | □ |

12 How do you store the test tubes immediately after sampling?

|  | Never | Seldom | Often | Always |
| --- | --- | --- | --- | --- |
| **a)** Lying on a workbench or other similar location | □ | □ | □ | □ |
| **b)** In the pocket of my work wear | □ | □ | □ | □ |
| **c)** In a test tube stand | □ | □ | □ | □ |
| **d)** By other mean:………………………………………………. | □ | □ | □ | □ |

The following questions ONLY concern sampling with paper based test requests

*It is important for you to mark ONE alternative for EACH row in the following section.*

13 How often does someone else mark the sampling time on the test request?

| Every day | Every week | Every month | More seldom | Never |
| --- | --- | --- | --- | --- |
| □ | □ | □ | □ | □ |

14 When do you mark the sampling time on the test request, if you do it yourself?

| I never mark sampling time | More than 30 min before sampling | 0-30 min before sampling | 0-30 min after sampling | More than 30 min after sampling |
| --- | --- | --- | --- | --- |
| □ | □ | □ | □ | □ |

15 How often do you perform the following tasks?

|  | Never | Seldom | Often | Always |
| --- | --- | --- | --- | --- |
| **a)** Compare the patient’s name and social security number with  the information on the test request | □ | □ | □ | □ |
| **b)** Use test requests that somebody else has filled in | □ | □ | □ | □ |
| **c)** Sign the test request | □ | □ | □ | □ |
| **d)** Check the information on the test request, if somebody else  has completed it | □ | □ | □ | □ |
| **e)** Adjust sampling time on the test request, if the marked time  differ with more than 30 min from the actual sampling time? | □ | □ | □ | □ |
| **f)** Check that the test request and test tube identification  (barcode) numbers match, before delivery to the laboratory | □ | □ | □ | □ |


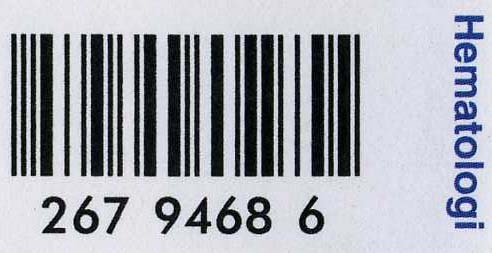

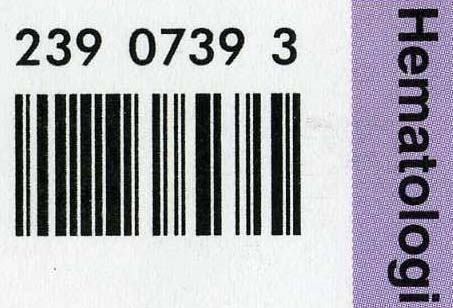


16 When do you label the test tube?

|  | Never | Seldom | Often | Always |
| --- | --- | --- | --- | --- |
| **a)** Before I approach the patient | □ | □ | □ | □ |
| **b)** Alongside the patient before sampling | □ | □ | □ | □ |
| **c)** Alongside the patient after sampling | □ | □ | □ | □ |
| **d)** At a later occasion | □ | □ | □ | □ |
| **e)** Somebody else has labelled the test tube in advance | □ | □ | □ | □ |
| **f)** Somebody else labels the test tube after sampling | □ | □ | □ | □ |

These final questions concern error reporting, ranking and suggestions

*It is important that you to mark ONE alternative for EACH row in the following section*

17 Approximately, how many error reports have you written after observing or making an error in venous blood sampling?

| Number of times | Haven’t written any |
| --- | --- |
| _ _ times | □ |

18 If you have refrained from writing a error report: What was/were the reason/reasons?

*(Please complete the questions below even if you have never written any report.)*

|  | Never | Seldom | Often | Always |
| --- | --- | --- | --- | --- |
| **a)** I don’t have enough time | □ | □ | □ | □ |
| **b)** It wouldn’t make any difference | □ | □ | □ | □ |
| **c)** Nobody else does | □ | □ | □ | □ |
| **d)** It is too complicated | □ | □ | □ | □ |
| **~~e)~~** ~~The head of the PHC writes the error reports~~ | ~~□~~ | ~~□~~ | ~~□~~ | ~~□~~ |
| **~~f)~~** ~~I am concerned about possible consequences~~  ~~(~~*~~specify below)~~* | ~~□~~ | ~~□~~ | ~~□~~ | ~~□~~ |
| **………………………………………………………………………………………………………………………** | | | | |
| **g)** By other reason:……………………………………………… | □ | □ | □ | □ |

19 To what extent do you agree in the following statements?

|  | Do not agree at all | Agree to some extent | Totally agree |
| --- | --- | --- | --- |
| **a)** *I have enough knowledge for my daily work with respect to*  *venous blood sampling and specimen handling* | □ | □ | □ |
| **b)** *Proper collection and handling of venous blood samples is*  *considered a priority at my PHC* | □ | □ | □ |

*Thank you for completing the questionnaire!*

*Please make sure that you have completed all questions, then place the questionnaire in the enclosed anonymous returning envelope, seal it, and give the envelope to the head of your PHC.*
